# Nitrogen Fixation in a Changing Arctic Ocean: An Overlooked Source of Nitrogen?

**DOI:** 10.3389/fmicb.2020.596426

**Published:** 2020-12-18

**Authors:** Lisa W. von Friesen, Lasse Riemann

**Affiliations:** Marine Biology Section, Department of Biology, University of Copenhagen, Helsingør, Denmark

**Keywords:** diazotroph, nifH, cyanobacteria, heterotrophic bacteria, climate change, primary production, marine, polar

## Abstract

The Arctic Ocean is the smallest ocean on Earth, yet estimated to play a substantial role as a global carbon sink. As climate change is rapidly changing fundamental components of the Arctic, it is of local and global importance to understand and predict consequences for its carbon dynamics. Primary production in the Arctic Ocean is often nitrogen-limited, and this is predicted to increase in some regions. It is therefore of critical interest that biological nitrogen fixation, a process where some bacteria and archaea termed diazotrophs convert nitrogen gas to bioavailable ammonia, has now been detected in the Arctic Ocean. Several studies report diverse and active diazotrophs on various temporal and spatial scales across the Arctic Ocean. Their ecology and biogeochemical impact remain poorly known, and nitrogen fixation is so far absent from models of primary production in the Arctic Ocean. The composition of the diazotroph community appears distinct from other oceans – challenging paradigms of function and regulation of nitrogen fixation. There is evidence of both symbiotic cyanobacterial nitrogen fixation and heterotrophic diazotrophy, but large regions are not yet sampled, and the sparse quantitative data hamper conclusive insights. Hence, it remains to be determined to what extent nitrogen fixation represents a hitherto overlooked source of new nitrogen to consider when predicting future productivity of the Arctic Ocean. Here, we discuss current knowledge on diazotroph distribution, composition, and activity in pelagic and sea ice-associated environments of the Arctic Ocean. Based on this, we identify gaps and outline pertinent research questions in the context of a climate change-influenced Arctic Ocean – with the aim of guiding and encouraging future research on nitrogen fixation in this region.

## Introduction

The Arctic Ocean (AO) covers only ~4% of the global ocean surface, but accounts for up to 10–14% of the total oceanic carbon dioxide sink (Bates and Mathis, 2009; Manizza et al., 2019). Therefore, with climate change proceeding at elevated speed in the Arctic region (Serreze et al., 2009; AMAP, 2019; Meredith et al., 2019), it is of importance for global carbon cycling to understand and predict current and future net primary production in the AO. Ongoing and predicted drastic changes in the AO include, e.g., decreasing area, thickness and age of sea ice (Stroeve and Notz, 2018), altered water column stratification (Peralta-Ferriz and Woodgate, 2015; Polyakov et al., 2020), rapid ocean acidification (Terhaar et al., 2020), increasing surface temperatures (Fyfe et al., 2013; Timmermans et al., 2017), rising discharge of freshwater (Terhaar et al., 2019), intensifying thaw of permafrost (Biskaborn et al., 2019), and large-scale hydrographical changes (e.g., Bluhm et al., 2015; Proshutinsky et al., 2015; Woodgate, 2018). The continuous thinning and withdrawal of sea ice stimulate pelagic and sympagic (sea ice-associated) primary production, but with nutrient availability – in particular nitrogen (N) – as a key determinant (Tremblay and Gagnon, 2009; Vancoppenolle et al., 2013; Arrigo and van Dijken, 2015; Fernández-Méndez et al., 2015; Lewis et al., 2020). In fact, the often prevalent N limitation of primary production in the AO (e.g., Codispoti et al., 2013; Tremblay et al., 2015; Mills et al., 2018) is predicted to intensify in some areas due to, e.g., increased stratification (Vancoppenolle et al., 2013; Slagstad et al., 2015). However, large regions – particularly in the eastern AO – are undersampled, and the many mechanisms regulating input and availability of N across the AO are intensively debated: e.g., turbulent nitrate fluxes (Randelhoff et al., 2020), advection of Pacific and Atlantic water (Lewis et al., 2020), glacial melt (Hopwood et al., 2020), riverine discharge (Terhaar et al., 2019), denitrification processes (Zeng et al., 2017), atmospheric deposition (Kühnel et al., 2013), shelf-break eddies (Watanabe et al., 2014), and photoammonification (Xie et al., 2012). Hence, accurate determination of sources and sinks of new N is a critical prerequisite for predictions of future net primary production and sequestration of carbon in the AO.

Diazotrophs are prokaryotes (bacteria and archaea) capable of converting inert gaseous dinitrogen (N_2_) to bioavailable ammonia in a process called biological nitrogen fixation (BNF; Postgate, 1970). Marine BNF has conventionally been attributed to photoautotrophic cyanobacteria (reviewed in Zehr, 2011), considered to be limited to relatively high-temperature (mainly ~ > 25°C), oligotrophic, photic waters of the tropical and subtropical parts of the global ocean (Stal, 2009; Sohm et al., 2011). There, BNF may support up to 50% of new production (Karl et al., 1997; Capone et al., 2005). However, in particular during the last decade, it has become evident that both cyanobacterial and non-cyanobacterial diazotrophs are more widely distributed and active in the global ocean than previously thought, including, e.g., low-temperature waters and coastal and upwelling areas (reviewed in Bombar et al., 2016; Zehr and Capone, 2020). These novel findings include the detection of BNF and diazotrophs in the AO (e.g., Blais et al., 2012; Fernández-Méndez et al., 2016; Shiozaki et al., 2018), thus setting a new scene for our understanding of N dynamics in the AO.

Here, we discuss current knowledge on diazotrophs and their activity in the pelagic and sympagic AO. We argue that BNF is a hitherto overlooked process and acquisition of basic knowledge on distribution, activity, and ecological drivers of diazotrophy is therefore imperative for analyses of N and carbon biogeochemistry in the current and future AO. We provide a set of pertinent research questions aiming to guide and inspire future research on diazotrophy in the AO, in particular in the light of climate change (Box 1).

## Widespread Nitrogen Fixation in the Arctic Ocean

**Figure 1:**
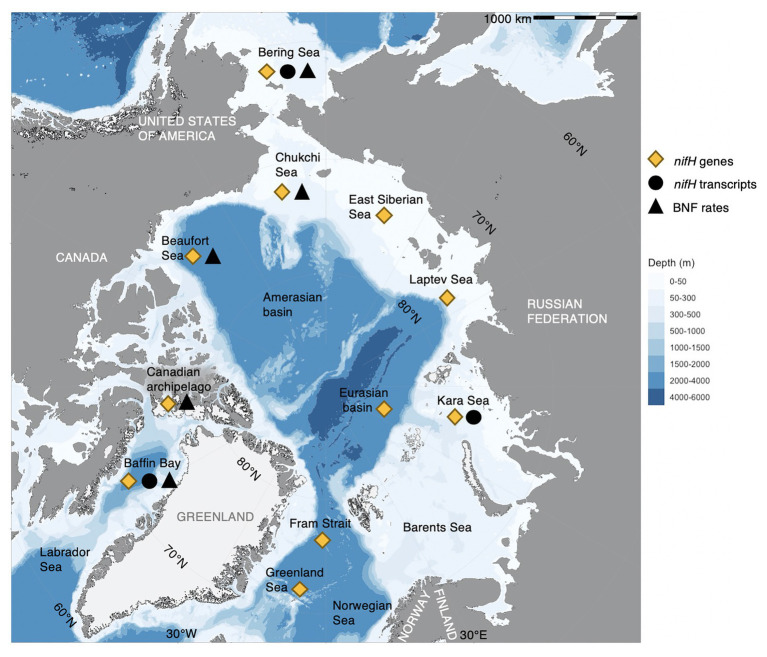
Biological nitrogen fixation (BNF) and diazotrophs across the Arctic Ocean. “*nifH* genes” refers to DNA-based detections (i.e., presence of *nifH*, putative diazotrophs), “*nifH* transcripts” to RNA-based detections (i.e., expression of *nifH*), and “BNF rates” to physiological rate measurements (i.e., quantitative measurement of nitrogen fixation, such as ^15^N_2_ incorporation). We infer BNF rates and *nifH* transcripts as confirmation of diazotrophy, marked black (triangle and circle, respectively), whereas *nifH* genes indicate a potential for diazotrophy, marked yellow (diamond). Findings are grouped by major Arctic regions (names in black) and do, therefore, not represent absolute geographical locations. For details on region, environment, depth, season, detected diazotrophs, *nifH* gene, and transcript abundance and BNF rates (when available) of the findings indicated in the map, see Supplementary Table S1. Data originate from Farnelid et al. (2011), Blais et al. (2012), Díez et al. (2012), Bowman et al. (2014), Fernández-Méndez et al. (2016), Shiozaki et al. (2017), Sipler et al. (2017), Harding et al. (2018), Shiozaki et al. (2018), and Salazar et al. (2019). Map produced in R (R Core Team, 2020) with the package ggOceanMaps (Vihtakari, 2020) based on bathymetry data from Amante and Eakins (2009) and glacier and land polygons from Natural Earth Data (www.naturalearthdata.com).

The AO, the smallest ocean on Earth (~14 million km^2^), is characterized by extensive shelf seas, sea ice, extreme seasonality, and major river and meltwater discharges – resulting in distinct water masses over heterogeneous shelves and deeper basins (Bluhm et al., 2015; Williams and Carmack, 2015). Inflow occurs from the adjacent Atlantic and Pacific Oceans over the Bering, Chukchi, and Barents shelves, with the only deep-water connection being through Fram Strait, which also holds the major outflow (Figure 1; Jakobsson et al., 2004). The AO is partly iron-rich (Klunder et al., 2012), which has been put forward as a potential advantage to, and regulator of, diazotrophs because of their high iron requirements (Blais et al., 2012; Shiozaki et al., 2017, 2018). Diazotrophs have by now been detected in pelagic and sympagic environments of the AO under wide-ranging environmental conditions (Figure 1; Supplementary Table S1), reaching from ice-free surface waters (e.g., Harding et al., 2018), estuaries (Blais et al., 2012; Sipler et al., 2017), and aphotic mesopelagic waters (Salazar et al., 2019), to sea ice brine (Díez et al., 2012), frost flowers (Bowman et al., 2014), sea ice melt-ponds, and algal aggregates (Fernández-Méndez et al., 2016). The environmental regulation of both cyanobacterial and non-cyanobacterial diazotrophs is emerging as more complex than previously thought (Zehr and Capone, 2020), which complicates the prediction of BNF in the AO – and in marine waters in general.

Diazotrophs are classically investigated by targeting the *nifH* gene encoding dinitrogenase reductase, a main protein responsible for BNF (Zehr et al., 2003). They are clustered based on nitrogenase gene phylogeny (Chien and Zinder, 1996), where Cluster I encompasses cyanobacteria and alpha-, beta-, and gamma-proteobacteria, Cluster II bacteria with an alternative nitrogenase and nitrogenases of methanogenic archaea, Cluster III anaerobic bacteria and archaea, and Cluster IV nonfunctional *nifH*-homologs of archaea (Zehr et al., 2003). Molecular data on diazotrophs in the AO include presence (DNA: putative diazotrophs, i.e., having the *nifH* gene; e.g., Fernández-Méndez et al., 2016) or expression of *nifH* genes (RNA; e.g., Shiozaki et al., 2017). DNA- and RNA-based detections are mostly *via* PCR amplicon sequencing using cloning/sequencing (Blais et al., 2012; Díez et al., 2012; Fernández-Méndez et al., 2016) or next-generation sequencing (e.g., Shiozaki et al., 2018), but metagenomic and metatranscriptomic approaches have also identified diazotrophs in the AO (Bowman et al., 2014; Salazar et al., 2019). Further, using ^15^N_2_ incorporation (Montoya et al., 1996), both bulk (e.g., Blais et al., 2012; Sipler et al., 2017) and cell-specific (Harding et al., 2018) BNF rates have been obtained from the AO. Despite limited geographical sampling coverage of the heterogeneous AO and the use of various molecular and physiological methodologies, the existing data collectively suggest a widespread distribution of diverse diazotrophs and that BNF may be of ecological importance.

## Potential Importance of Nitrogen Fixation in the Arctic Ocean

By combining BNF rates from the Beaufort Sea and northern Baffin Bay (Blais et al., 2012) with new measurements from the Chukchi Sea, Sipler et al. (2017) estimated that if BNF of similar magnitude occurs in surface waters of ice-free shelves across the AO in summer (June–September), it would account for a N input of up to 3.5 ± 0.7 Tg N y^−1^ – representing ~2.7% of a global BNF estimate. Albeit based on sparse data, it proposes BNF to influence the N budget of the AO (Sipler et al., 2017). However, estimates on the importance of BNF vary across the AO: ranging from stimulation of up to 0.89% of new primary production in the Bering Sea (Shiozaki et al., 2017), 4.3% in the Beaufort Sea (Blais et al., 2012), 7.0% in the central AO (Fernández-Méndez et al., 2016), to occasionally 17% in the Chukchi Sea (Shiozaki et al., 2018). Considering the drastic effect BNF could have on primary production by alleviating N limitation, BNF measurements should – when available in sufficient number and quality – be incorporated into models of current and future net primary production across the AO region. However, to our knowledge, BNF has not yet been included as a N source in such models of the AO system (e.g., Earth system models, Vancoppenolle et al., 2013; SINMOD, Slagstad et al., 2015; ECCO2-Darwin, Manizza et al., 2019) because diazotrophs have been parameterized not to occur under such environmental conditions. This is in contrast to a global model of diazotroph distribution predicting the presence of diazotrophs in the AO based on most recent knowledge of how environmental regulation varies between groups (Tang and Cassar, 2019). Hence, accumulating evidence suggests that BNF may be an overlooked source of new N in the AO, hampering the understanding and prediction of productivity and carbon flux in the Arctic.

BOX 1 **Nitrogen fixation in a changing Arctic Ocean: Avenues for future research**

**Figure 2:**
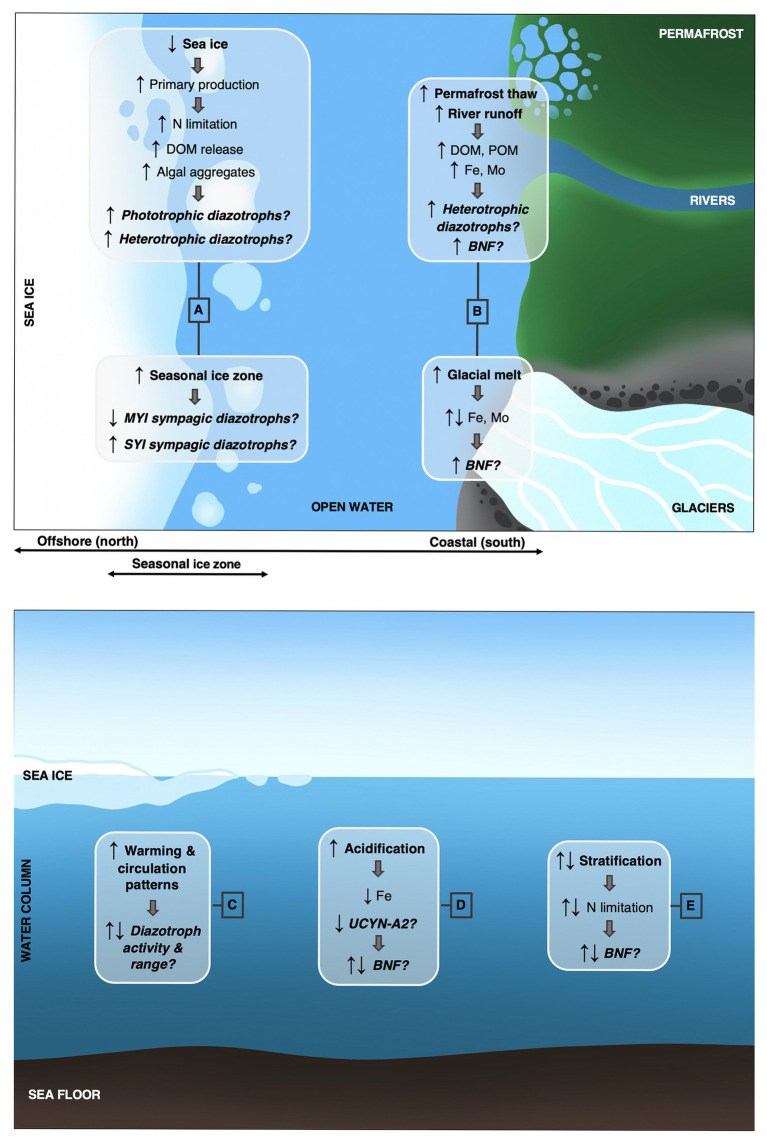
Conceptual schematic in (top) birds-eye and (bottom) cut-through perspective of current and predicted environmental changes in the Arctic Ocean with potential implications for diazotrophs and biological nitrogen fixation. See text of (Box 1) for elaborated explanations of changes and potential responses related to **(A)** sea ice, **(B)** river runoff, permafrost thaw and glacial melt, **(C)** ocean warming and circulation patterns, **(D)** ocean acidification, and **(E)** stratification. Arrows depict directional change and/or competitive advantage/disadvantage. MYI, multiyear ice; SYI, single-year ice; DOM, dissolved organic matter; POM, particulate organic matter; Fe, iron; Mo, molybdenum; N, nitrogen; BNF, biological nitrogen fixation; UCYN-A, Candidatus *Atelocyanobacterium thalassa*.

The manifold changes induced by climate change inevitably alter abiotic and biotic conditions surrounding organisms (AMAP, 2019; Meredith et al., 2019) and will impact diazotrophy (Wrightson and Tagliabue, 2020). The changes considered to be most directly relevant to diazotrophs and BNF in the AO are here discussed and research questions outlined (Figure 2). As the composition of diazotrophs in the AO encompasses functionally diverse organisms, e.g., autotrophs and (photo-)heterotrophs, symbionts, and free-living cells, and potentially associated to particles/aggregates or sea ice, the responses to environmental changes and implications for BNF are expectedly group-specific and multifaceted.

**A**: Sea ice reductions entail an increasing seasonal ice zone and ultimate replacement of multiyear ice (MYI) with single-year ice (SYI) (Stroeve and Notz, 2018), with consequences for sympagic biodiversity (Vincent, 2010; Hop et al., 2020). *Are some diazotrophs reliant on MYI and/or SYI habitats, and which are thus the biogeochemical consequences when SYI is expanding on behalf of MYI?*

The changing light regime stimulates pelagic and sympagic primary production on different scales (Fernández-Méndez et al., 2015; Clement Kinney et al., 2020; Lewis et al., 2020) and will likely be coupled to increased N consumption. *How may increasing N limitation favor BNF in general and increasing light availability influence phototrophic diazotrophs in particular?*

Increasing primary production generates more dissolved organic matter (DOM) which, depending on the quantity and quality of DOM, is thought to stimulate heterotrophic (reviewed in Bombar et al., 2016) and potentially mixotrophic diazotrophs (Benavides et al., 2020). *How may increasing DOM stimulate heterotrophic and mixotrophic BNF?*

Low oxygen microenvironments are proposed sites of BNF by heterotrophic bacteria (Paerl, 1985; Riemann et al., 2010), which in the AO could be, e.g., sea ice (Rysgaard et al., 2008) and algal aggregates (Fernández-Méndez et al., 2014). *May the changing sea ice conditions and/or increasing levels of particulate/aggregate matter due to elevated pelagic and sympagic primary production provide such low-oxygen loci and stimulate heterotrophic BNF?*

**B**: Freshwater input from rivers, permafrost thaw, and glaciers is increasing in the AO (Mouginot et al., 2019; Terhaar et al., 2019) and may affect levels of trace metals (e.g., iron and molybdenum), DOM and particulate organic matter (POM) (Holmes et al., 2012; Hopwood et al., 2020; Michaud et al., 2020). *To what extent may trace metals supplied by glacial melt and river runoff stimulate BNF? How may remobilization of DOM and POM from permafrost stimulate heterotrophic and mixotrophic BNF?*

**C**: Ocean warming (Timmermans et al., 2017) has direct and/or indirect effects on microbes of the N cycle, here among diazotrophs (Levitan et al., 2010; Fu et al., 2014). Temperature regulation is known to vary between diazotroph groups (Sohm et al., 2011). In the AO, increasing temperature might impact estuarine and marine waters differently in terms of BNF potential (Blais et al., 2012). *How will increasing temperatures in the AO affect growth rates and BNF potential of diazotrophs?*

Ultimately, warming and altered circulation patterns (e.g., Bluhm et al., 2015; Woodgate, 2018) may lead to range contraction and/or expansion for diazotrophs (Sherwood et al., 2014; Cabello et al., 2020), depending on respective autecology. *How will warming and circulation changes affect the biogeography of diazotrophs,* e.g., *to what extent may northward spreading of warmer-water diazotrophs and/or habitat contraction for potentially cold-adapted diazotrophs alter community composition and BNF activity?*

**D**: Ocean acidification alters the carbonate system (Terhaar et al., 2020), stimulating some diazotrophs while suppressing others (Eichner et al., 2014; Luo et al., 2019). *What group-specific responses of diazotrophs can be expected in the AO*, e.g., *will increased undersaturation of calcite selectively affect the diazotroph UCYN-A2 due to the coccolithophore host having a calcifying life-stage* (Thompson et al., 2014; Cabello et al., 2020)?

Ocean acidification can lower the bioavailability of iron (Shi et al., 2010), a key regulating nutrient for diazotrophs (Sohm et al., 2011). *How may this come to hamper BNF in the currently partly iron-rich AO?*

**E**: Stratification is increasing in the AO (Peralta-Ferriz and Woodgate, 2015), but in some regions also decreasing (Polyakov et al., 2020) – likely causing increasing/decreasing N limitation on various temporal and spatial scales. Non-cyanobacterial diazotrophs seem less down-regulated in N-replete waters than do their cyanobacterial counterparts (Knapp, 2012; Bombar et al., 2016; Moisander et al., 2017), but the sensitivity of BNF to fixed N is overall emerging as more complex than previously thought – also for cyanobacteria (Farnelid et al., 2016; Zehr and Capone, 2020). *Will increasing N limitation in parts of the AO provide a competitive advantage to diazotrophs? Will changing N availabilities alter the relative abundance of phototrophic and heterotrophic diazotrophs?*

## Cyanobacterial Diazotrophs May be of Higher Relative Abundance at Inflow and Outflow Shelves

Relatives of well-known cyanobacterial diazotrophs from lower latitudes, e.g., Nostocales (Blais et al., 2012; Díez et al., 2012; Fernández-Méndez et al., 2016), *Trichodesmium* spp. and Chroococcales (Díez et al., 2012), have only been sporadically detected in the AO. Along a transect from the North Pacific into the Bering Sea, the diazotroph community drastically changed – where all studied cyanobacterial diazotrophs, except one, had disappeared in the Bering Sea (Shiozaki et al., 2017). The one detected actively expressing *nifH* is a symbiosis between the unicellular photoheterotrophic N_2_ fixing cyanobacterium Candidatus *Atelocyanobacterium thalassa* (UCYN-A, Cluster I member) and eukaryotic photosynthetic algae. It has now, contrary to previous views, been detected in marine waters worldwide (Farnelid et al., 2016). Recently, UCYN-A was again identified in mainly coastal surface waters of the Bering (especially sublineage A1) and Chukchi Seas (especially sublineage A2) (Harding et al., 2018; Shiozaki et al., 2018). Interestingly, UCYN-A cell-specific BNF rates accounted for bulk BNF rates in the Bering Sea and were of similar magnitude as in lower-latitude warm water locations. In the Chukchi Sea, however, UCYN-A was less abundant and did not account for bulk BNF rates (Harding et al., 2018). This indicates that other diazotrophs contributed to BNF. On the Atlantic side of the AO, diverse putative cyanobacterial diazotrophs were identified in sea ice brine from the Fram Strait and seawater from the Greenland Sea (Díez et al., 2012). This study was, however, based on small clone libraries and applied different primers than other AO studies. Taken together, the available but sparse data indicate that cyanobacterial diazotrophs may be of higher relative abundance at the inflow and outflow shelves (Díez et al., 2012; Shiozaki et al., 2017, 2018; Harding et al., 2018) compared to inner parts of the AO. This is possibly due to the strong interconnection with the adjacent Atlantic (Fernández-Méndez et al., 2016) and Pacific Oceans (Shiozaki et al., 2018) – but the relative role of advection versus endemic populations remains elusive (Harding et al., 2018). Clearly, composition and occurrence of cyanobacterial diazotrophs in the AO, of which UCYN-A is the most frequently detected, differ both between the internal seas and relative to other oceans.

## Predominance of Non-Cyanobacterial Diazotrophs in the Arctic Ocean

In the central eastern AO, the Eurasian basin, analysis of small clone libraries from water and sea ice revealed an overall dominance of putative non-cyanobacterial diazotrophs from Cluster I, and high relative abundances of Cluster III in melt ponds and algal aggregates therein (Fernández-Méndez et al., 2016). The Eurasian basin community showed a distinct composition when compared to *nifH* gene sequences from other Arctic seas, polar habitats (Arctic tundra, Antarctic microbial mats), and boreal/subtropical Atlantic waters (Fernández-Méndez et al., 2016). On a larger scale, dominance of non-cyanobacterial Cluster III genes, as inferred from deep *nifH* amplicon sequencing, clearly distinguished the Arctic (represented by a location in Baffin Bay) from eight other distinct biogeographical regions around the global ocean (Farnelid et al., 2011). In a fjord of Baffin Bay and in the Mackenzie river plume (Beaufort Sea), Cluster III interestingly dominated at the locations showing highest BNF rates – including stations where no cyanobacteria were detected among the *nifH* clones (Blais et al., 2012). Similarly, in the Chukchi Sea, greater than 80% of retrieved *nifH* sequences were affiliated with Cluster III, and a complex vertical pattern of BNF rates suggests the presence of nonphototrophic diazotrophy (Shiozaki et al., 2018). Further studies are needed to elucidate the relative contribution of different diazotrophs to BNF. Detection of BNF rates and *nifH* transcripts in aphotic waters can conceivably be attributed to non-cyanobacterial diazotrophy (Moisander et al., 2017; Benavides et al., 2018), and notably, *nifH* transcription by a heterotrophic diazotroph (as inferred from metatranscriptomics paired with a metagenome assembled genome) was recently detected in mesopelagic waters of Baffin Bay (Salazar et al., 2019). That study moreover detected *nifH* transcripts in aphotic waters of the Kara Sea and *nifH* genes in aphotic waters of the Greenland and Laptev Seas. There are thus indications of aphotic, plausibly non-cyanobacterial, diazotrophy in the AO. With the numerous dark environments due to sea ice cover, season, or depth, it may be an important avenue for future research. Taken together, the available data indicate that non-cyanobacterial diazotrophy, likely attributable to heterotrophic bacteria, may be ecologically important in the AO.

## Conclusion

There are strong indications of cyanobacterial and non-cyanobacterial diazotrophy by unique, occasionally active communities across environments in the AO. It suggests that BNF may have local to large-scale consequences for N dynamics and carbon flux in the AO, but likely with large variation between regions. As the data are sparse and vast uncharted regions remain, it is ambiguous to what degree, and under which conditions, diazotrophs influence N availability and ocean productivity. Therefore, coming research should aim to cover wider temporal, geographical, vertical, and environmental scales to ultimately discern the ecological role of diazotrophs in the rapidly changing AO. It will be important to acknowledge the highly heterogeneous Arctic environment and to direct efforts toward often undersampled regions such as inner shelf seas and the central basins. Future environmental perturbations caused by climate change will likely have multiple implications for BNF (Box 1) – stressing the need for acquisition of baseline data on BNF magnitude and on the distribution, diversity, function, regulation, and the potential ecosystem impact of diazotrophs.

## Author Contributions

LvF wrote the first version of the manuscript and both authors carried out subsequent revisions. Both authors contributed to the article and approved the submitted version.

### Conflict of Interest

The authors declare that the research was conducted in the absence of any commercial or financial relationships that could be construed as a potential conflict of interest.
