# Supplementary material for: Nitrogen Fixation in a Changing Arctic Ocean: An Overlooked Source of Nitrogen?
Source: Front Microbiol. 2020 Dec 18;11:596426. doi: 10.3389/fmicb.2020.596426 (PMC7775723; doi:10.3389/fmicb.2020.596426)
Supplement: Supplementary file 1 [file Table_1.docx]

Supplementary Material

**Table S1.** Compilation of studies reporting detection of putative diazotrophs (DNA-based detection of *nifH*), *nifH* transcripts (RNA-based detection) and/or measurements of nitrogen fixation rates in various regions, environments, depths and seasons across the Arctic Ocean.


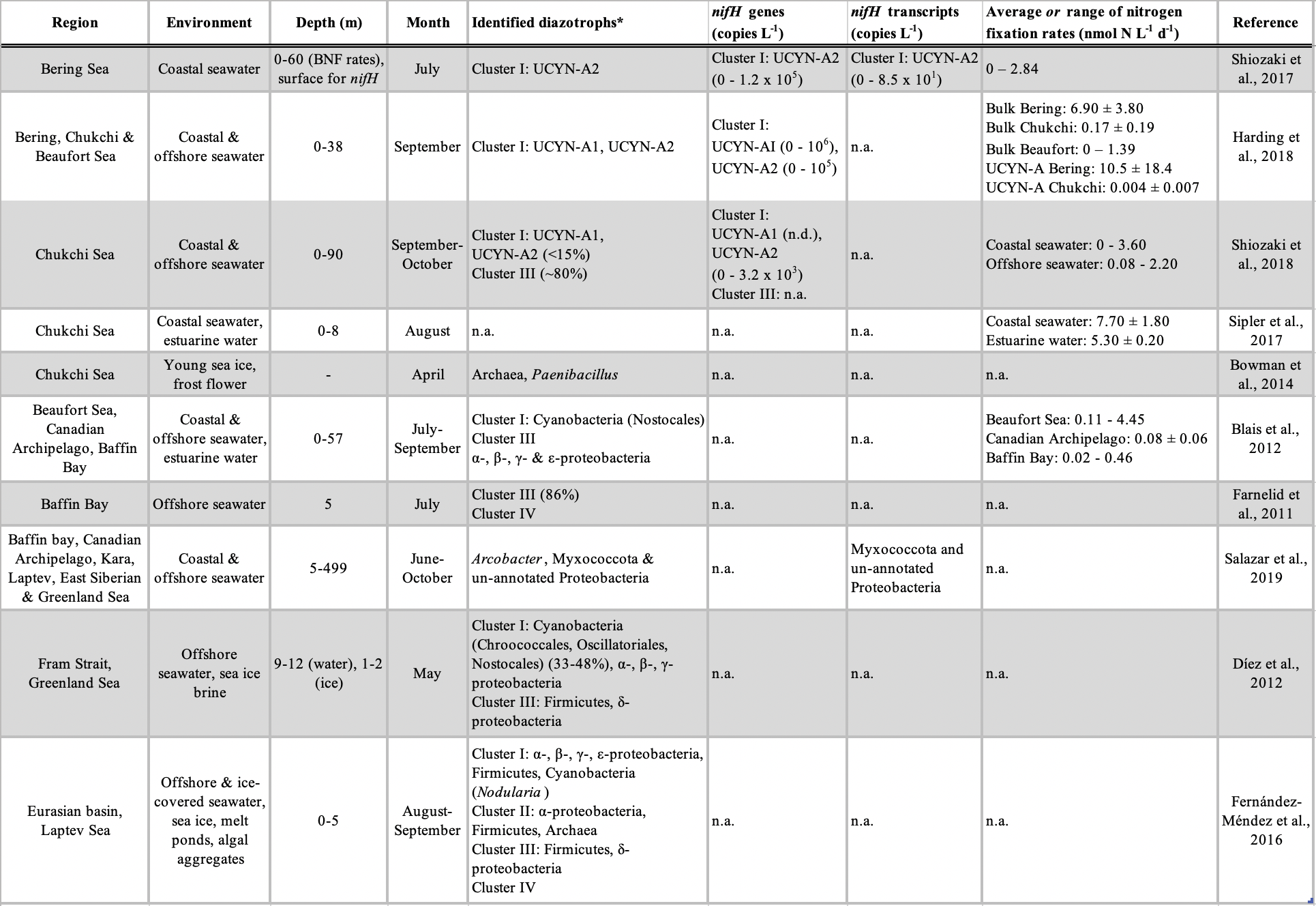

Geographical regions are depicted in Figure 1. *nifH* encodes dinitrogenase reductase.
n.a. = not analyzed, n.d. = not detected.
*percentages of relative abundances are presented in parentheses, and cluster-annotations of identified diazotrophs specified, when stated in the original study.

# References

Blais, M., Tremblay, J., Jungblut, A. D., Gagnon, J., Martin, J., Thaler, M., et al. (2012). Nitrogen fixation and identification of potential diazotrophs in the Canadian Arctic. *Global Biogeochem. Cycles* 26, GB3022. doi:10.1029/2011GB004096.

Bowman, J. S., Berthiaume, C. T., Armbrust, E. V., and Deming, J. W. (2014). The genetic potential for key biogeochemical processes in Arctic frost flowers and young sea ice revealed by metagenomic analysis. *FEMS Microbiol. Ecol.* 89, 376–387. doi:10.1111/1574-6941.12331.

Díez, B., Bergman, B., Pedrós-Alió, C., Antó, M., and Snoeijs, P. (2012). High cyanobacterial *nifH* gene diversity in Arctic seawater and sea ice brine. *Environ. Microbiol. Rep.* 4, 360–366. doi:10.1111/j.1758-2229.2012.00343.x.

Farnelid, H., Andersson, A. F., Bertilsson, S., Al-Soud, W. A., Hansen, L. H., Sørensen, S., et al. (2011). Nitrogenase gene amplicons from global marine surface waters are dominated by genes of non-cyanobacteria. *PLoS One* 6, e19223. doi:10.1371/journal.pone.0019223.

Fernández-Méndez, M., Turk-Kubo, K. A., Buttigieg, P. L., Rapp, J. Z., Krumpen, T., Zehr, J. P., et al. (2016). Diazotroph diversity in the sea ice, melt ponds, and surface waters of the Eurasian Basin of the Central Arctic Ocean. *Front. Microbiol.* 7, 1–18. doi:10.3389/fmicb.2016.01884.

Harding, K., Turk-Kubo, K. A., Sipler, R. E., Mills, M. M., Bronk, D. A., and Zehr, J. P. (2018). Symbiotic unicellular cyanobacteria fix nitrogen in the Arctic Ocean. *Proc. Natl. Acad. Sci.* 115, 13371–13375. doi:10.1073/pnas.1813658115.

Salazar, G., Paoli, L., Alberti, A., Huerta-Cepas, J., Ruscheweyh, H.-J., Cuenca, M., et al. (2019). Gene expression changes and community turnover differentially shape the global ocean metatranscriptome. *Cell* 179, 1068–1083. doi:10.1016/j.cell.2019.10.014.

Shiozaki, T., Bombar, D., Riemann, L., Hashihama, F., Takeda, S., Yamaguchi, T., et al. (2017). Basin scale variability of active diazotrophs and nitrogen fixation in the North Pacific, from the tropics to the subarctic Bering Sea. *Global Biogeochem. Cycles* 31, 996–1009. doi:10.1002/2017GB005681.

Shiozaki, T., Fujiwara, A., Ijichi, M., Harada, N., Nishino, S., Nishi, S., et al. (2018). Diazotroph community structure and the role of nitrogen fixation in the nitrogen cycle in the Chukchi Sea (western Arctic Ocean). *Limnol. Oceanogr.* 63, 2191–2205. doi:10.1002/lno.10933.

Sipler, R. E., Gong, D., Baer, S. E., Sanderson, M. P., Roberts, Q. N., Mulholland, M. R., et al. (2017). Preliminary estimates of the contribution of Arctic nitrogen fixation to the global nitrogen budget. *Limnol. Oceanogr. Lett.* 2, 159–166. doi:10.1002/lol2.10046.
